# Reading discordant bedside indices in sepsis-induced cardiomyopathy: an evidence-mapping framework for diagnosis and trial design

**DOI:** 10.3389/fcvm.2026.1918976

**Published:** 2026-09-29

**Authors:** Lulu Zhang, Siye Zhang

**Affiliations:** Department of Intensive Care Unit, Hunan Provincial People’s Hospital (The First Affiliated Hospital of Hunan Normal University), Changsha, China

**Keywords:** clinical decision-making, construct validity, echocardiography, right ventricular dysfunction, sepsis-induced cardiomyopathy, ventriculo-arterial coupling

## Abstract

Sepsis-induced cardiomyopathy (SICM) affects 20%–30% of patients with septic shock and independently predicts mortality, yet it lacks a consensus definition or validated reference standard. Bedside diagnostic indices—left ventricular ejection fraction (LVEF), global longitudinal strain, E/e′, troponin level, and cardiac index—are frequently discordant, and no principled framework exists for interpreting this discordance. In parallel, every major pharmacological intervention targeting SICM, including levosimendan, corticosteroids, and beta-blockers, has failed to demonstrate consistent mortality benefit across randomised trials. Herein, we propose that both impasses may share a single root cause: a construct validity failure in which bedside indices used for diagnosis and trial enrolment do not reliably identify the specific pathophysiological processes that they are assumed to represent. We present a structured evidence-mapping framework that organises six bedside diagnostic methods against seven pathophysiological trait dimensions of SICM, grading the evidence for each combination against explicit criteria and identifying structural blind spots. The framework explains why index discordance is a diagnostic signal rather than a measurement error, identifies two dimensions—metabolic dysfunction and cardiac-specific inflammatory signalling—that are structurally invisible to all current bedside diagnostics and whose invisibility offers a testable explanation for repeated trial failures, and translates these insights into a three-step bedside reasoning framework. When LVEF is depressed but longitudinal function is preserved, the framework directs assessment towards ventriculo-arterial uncoupling rather than inotrope escalation; when right ventricular dysfunction predominates, it directs assessment towards reverse resuscitation. The framework reframes index discordance from a source of diagnostic paralysis into a source of clinical precision and defines the enrolment biomarker agenda required to make future SICM trials mechanistically interpretable.

## 1 Introduction

A 68-year-old man is admitted to the intensive care unit in a state of septic shock. Norepinephrine has been uptitrated from 0.1 to 0.3 μg/kg/min over the past 2 h. Bedside echocardiography shows a left ventricular ejection fraction (LVEF) of 40% and a mitral annular plane systolic excursion (MAPSE) of 12 mm. The cardiac index (CI) measured via pulse contour analysis is 2.8 L/min/m^2^. The nurse asks, “Should we start dobutamine?”

Three indices. Two answers.

LVEF says yes: 40% is depressed, and this patient has systolic dysfunction. MAPSE says not so fast: 12 mm sits at the lower limit of normal, suggesting that longitudinal fibre shortening at the base is relatively preserved despite the low ejection fraction. CI says borderline: 2.8 L/min/m^2^ may represent relative adequacy or relative failure depending on the metabolic demand. LVEF and MAPSE are pointing in different directions. In a patient whose vasopressor requirement has just tripled, that discordance is not a measurement problem. It is a physiological signal, and without a framework for reading it, the clinician is left guessing.

Sepsis-induced cardiomyopathy (SICM) affects approximately 20%–30% of patients with septic shock and independently predicts mortality (1–4). It is characterised by reversible, non-ischaemic biventricular dysfunction. Systolic function typically recovers within days to 1 week in survivors, while diastolic dysfunction may resolve more slowly (5). Yet, despite decades of investigation, SICM remains one of the most diagnostically contested entities in critical care. It has no consensus definition and no validated reference standard. Without a validated reference standard, no single index can claim diagnostic primacy, and their disagreements cannot be adjudicated by appeal to an external criterion. The diagnostic indices that clinicians use daily—LVEF, global longitudinal strain (GLS), E/e′, troponin, and cardiac index—are not interchangeable measurements of a single construct. They capture different pathophysiological dimensions of a multifaceted syndrome, and their disagreement reflects genuine biological heterogeneity and not measurement error.

This diagnostic impasse has a clinical twin: a therapeutic impasse of equal magnitude. Over the past two decades, every major pharmacological intervention targeting SICM has produced inconsistent trial results. Levosimendan—evaluated in more than 10 randomised controlled trials (RCTs)—showed mortality benefit in early single-centre studies (6) but failed to replicate the benefit in larger analyses, with the most comprehensive meta-analyses reporting no survival advantage over dobutamine (7, 8). Corticosteroids, across 45 RCTs that enrolled over 9,500 patients, reduced short-term mortality by a statistically marginal risk ratio of 0.93—a signal so heterogeneous that its clinical meaning remains actively debated (9). Beta-blockers, including esmolol and landiolol, showed mortality benefit in some single-centre trials (10) but neutral or potentially harmful effects in multicentre RCTs; the STRESS-L trial was terminated early after an interim analysis showed that landiolol was unlikely to benefit and might cause harm (11).

The standard explanation for these failures is statistical: underpowered trials, heterogeneous populations, and inconsistent endpoints. We propose, as a complementary hypothesis, a more fundamental explanation rooted in construct validity. The bedside indices used to enrol patients do not reliably identify the specific pathophysiological processes that those interventions are designed to address. LVEF depression is not equivalent to the presence of any specific pathophysiological process: the same LVEF value can arise from true contractile failure, volume-loading geometrical dilution, afterload-driven E_a_/E_es_ deterioration, or ventriculo-arterial (VA) uncoupling. Meanwhile, the dimensions that anti-inflammatory and metabolic support agents would need to reach—cardiac-specific inflammatory signalling and intracellular energetic failure—have no bedside measurement equivalent. They are structurally invisible to standard monitoring. The result is systematic construct dilution: patients without the target pathophysiology are randomly assigned to treatment arms that were originally designed for patients who have it, the signal is diluted, and the trial fails. The problem is then not only statistical but also structural: SICM has never been formally defined as a multidimensional construct.

This review applies the logic of construct validation—an established methodology for validating measurements in the absence of a reference standard (12)—to map six bedside diagnostic methods against seven pathophysiological trait dimensions of SICM, grading each combination against explicit criteria (Section 2.1). The resulting framework does three things: it explains why bedside index discordance is a feature rather than a flaw, it identifies the structural blind spots that may help explain why therapeutic trials have consistently failed, and it translates both into a practical bedside reasoning framework that transforms index discordance from a source of clinical paralysis into a source of diagnostic precision. This framework does not require new tools. It requires a new way of reading the tools already at the bedside.

## 2 The evidence-mapping framework

### 2.1 Constructing the evidence map

We organised six bedside diagnostic methods—transthoracic echocardiography (TTE; M1), speckle-tracking echocardiography (STE; M2), cardiac magnetic resonance (CMR; M3), circulating biomarkers (M4), invasive haemodynamic monitoring by pulmonary artery catheter or transpulmonary thermodilution with pulse-contour analysis (PAC/PiCCO; M5), and electrocardiography (ECG; M6)—against seven pathophysiological trait dimensions of SICM into a structured evidence matrix (Figure 1). The approach adapts the construct validation logic previously explained by Campbell and Fiske for validating measurements when no gold standard exists (12). M2 is a near-bedside, rather than a bedside, method—speckle-tracking strain requires offline post-processing and cannot be repeated at short intervals—therefore, MAPSE or tissue-Doppler s′ is the bedside longitudinal index in this framework and GLS is the confirmatory one.

**Figure 1:**
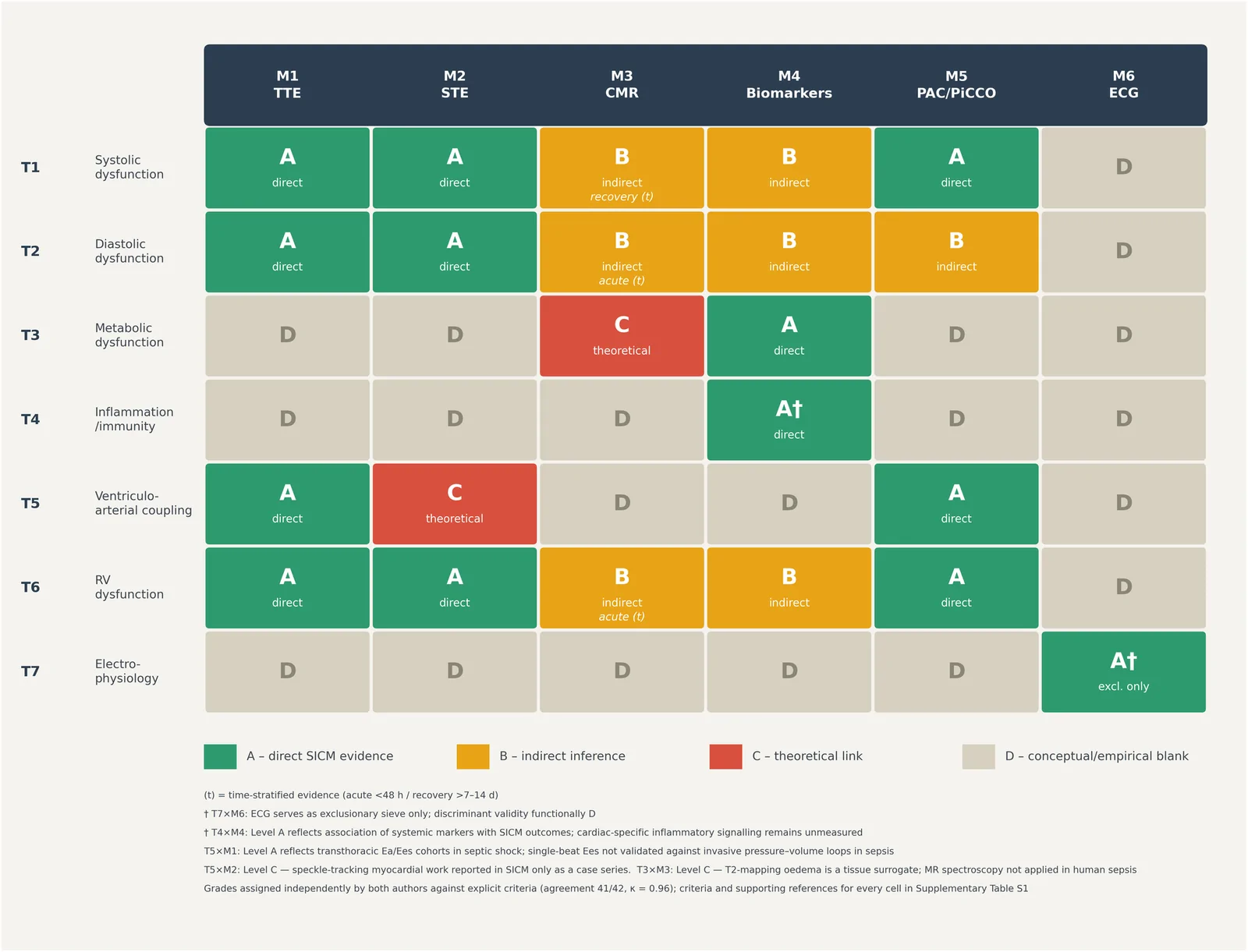
An evidence-mapping matrix for sepsis-induced cardiomyopathy. The rows represent seven pathophysiological trait dimensions (T1–T7), and the columns represent six bedside diagnostic methods (M1–M6). Evidence grades: A, direct evidence from sepsis or septic shock populations; B, indirect inference with biologically plausible extension; C, theoretical link without cohort data in sepsis; and D, conceptual or empirical blank. (t), time-stratified evidence differing between acute (<48 h) and recovery (>7–14 days) phases. †T4 × M4: level A reflects systemic inflammatory marker association with SICM outcomes; cardiac-specific signalling remains unmeasurable at the bedside. †T7 × M6: ECG serves only as an exclusionary sieve; discriminant validity is functionally D. T5 × M1: level A reflects transthoracic E_a_/E_es_ cohorts in septic shock; the single-beat E_es_ estimate has not been validated against invasive pressure-volume loops in sepsis. T5 × M2: level C reflects case-level reports of speckle-tracking myocardial work only. Grades were assigned independently by both authors against explicit criteria (agreement in 41/42, *κ* = 0.96); the criterion applied and the supporting references for every cell are given in Supplementary Table S1.

The seven dimensions were derived from the mechanistic literature on SICM (1–3, 13) using three criteria: a property of the myocardium or of its coupling to the circulation, separable from the others in the sense of being measured (or not measured) by different methods rather than being causally independent—T3 and T4 are upstream of the mechanical dimensions—and at least, in principle, measurable by one of the six methods. Microcirculatory dysfunction (a systemic vascular process whose cardiac consequences—impaired substrate delivery and endothelial-leukocyte activation—are captured in T3 and T4) and autonomic dysfunction (a modulator of T5 and T7 without a cardiac-specific index) were therefore not given separate rows.

Evidence for each cell was identified through iterative PubMed/MEDLINE and Embase searches (to June 2026) combining sepsis terms with terms for each method and trait, which was supplemented by hand searching; adult human studies were prioritised. The map is a structured narrative synthesis rather than a systematic review.

#### 2.1.1 Grading criteria

Each trait-method cell was graded A (direct evidence: at least one clinical study in adult sepsis or septic shock in which the method measured an index of the trait and reported prevalence, diagnostic performance, or an outcome association), B (indirect inference: the method measures a relevant index, but the evidence comes from a different disease phase, from non-sepsis or animal populations, or from a surrogate not validated for the trait in sepsis), C (theoretical link: a measurement pathway is proposed, but no cohort measurement exists in sepsis), or D (blank: D-conceptual where the method cannot measure the trait in principle; D-empirical where measurement is conceivable but no sepsis data exist); time stratification was done where evidence differed between the acute (<48 h) and recovery (>7–14 days) phases. Both authors graded all 42 cells independently against these criteria; agreement was reached in 41 of 42 cells (Cohen's *κ* = 0.96), and the discordant cell (T3 × M3) was resolved by consensus as C. The map contains 13 A, 7 B, 2 C, and 20 D cells; the grade, criterion, and supporting references for every cell are given in Supplementary Table S1.

The framework rests on three interpretive principles. Convergent validity (multiple independent methods agreeing on the same trait) raises diagnostic confidence. Structural isolation (a trait accessible by only one method or none) identifies a bedside blind spot. Discriminant validity (two methods disagreeing about the same patient) localises genuine pathophysiological heterogeneity rather than measurement error. The clinical value of index discordance lies not in resolving it by choosing the better instrument but in reading what it reveals about which trait dimensions are engaged. The three-step bedside reasoning framework operationalising these principles is presented in Figure 2B.

**Figure 2:**
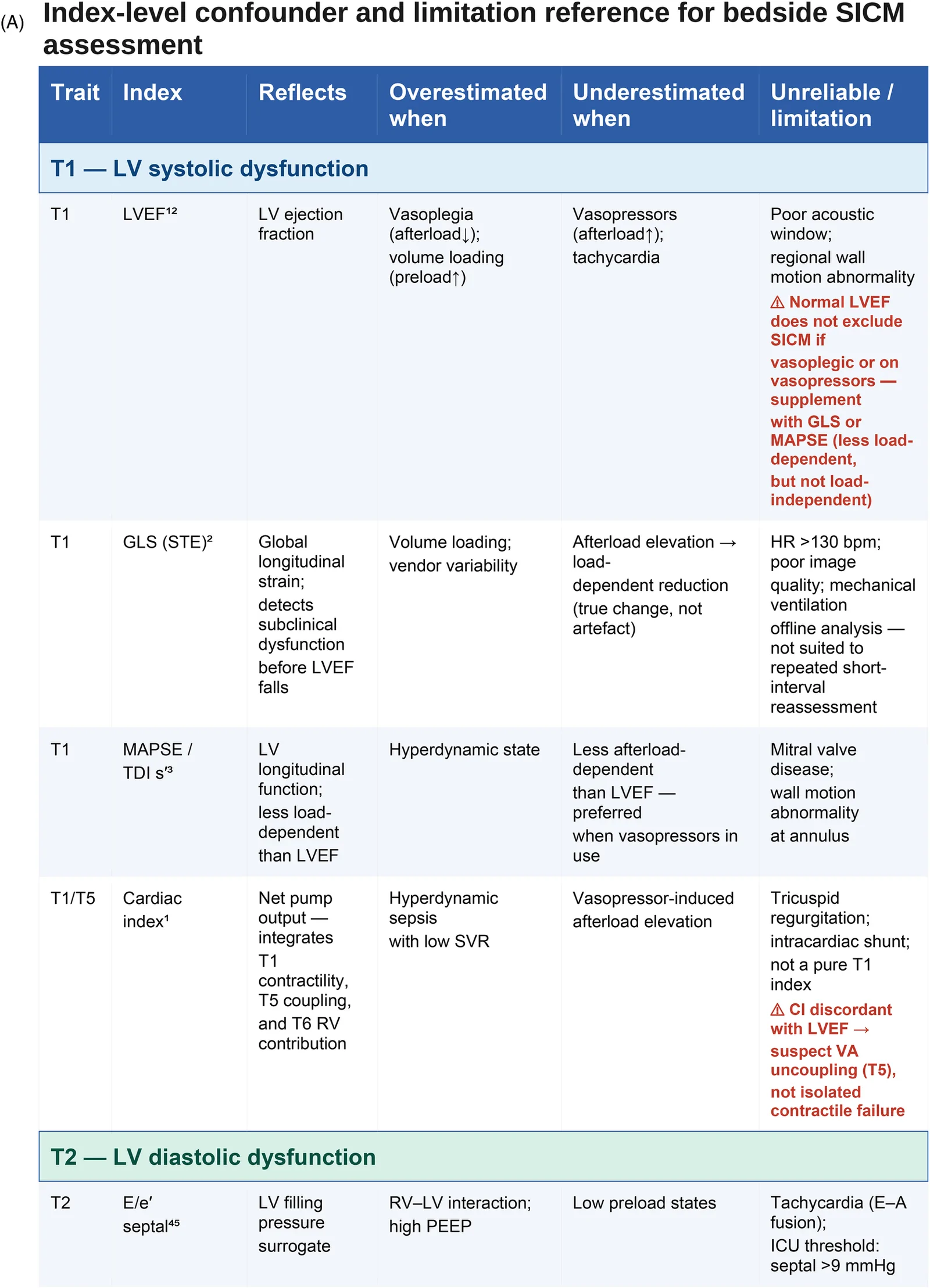
**(A)** Index-level confounder and limitation reference for bedside SICM assessment. For each pathophysiological dimension and its corresponding bedside index, the table specifies what the index reflects, the conditions under which it overestimates or underestimates dysfunction, and key technical limitations. This reference table operationalises the loading-condition and technical confounders discussed throughout Section 2 and provides a side-by-side comparison of echocardiographic indices (LVEF, GLS, MAPSE/TDI s’, E/e′, TAPSE, RVFWLS, and RVEDA/LVEDA) in terms of what each reflects, their load dependence, and their limitations. **(B)** A three-step framework for interpreting discordant bedside indices in SICM. Step 1 identifies the discordant index pair. Step 2 excludes the methodological artefact by reference to the confounder table **(A)**. Step 3 translates the result into clinical action: when the artefact is identified, the less-confounded index is trusted, while when genuine trait heterogeneity is confirmed, the multidimensional phenotype is mapped and assessment escalated before intervention. The key principle is that a mixed phenotype profile is not a diagnostic failure but a source of diagnostic precision.

**Figure:**
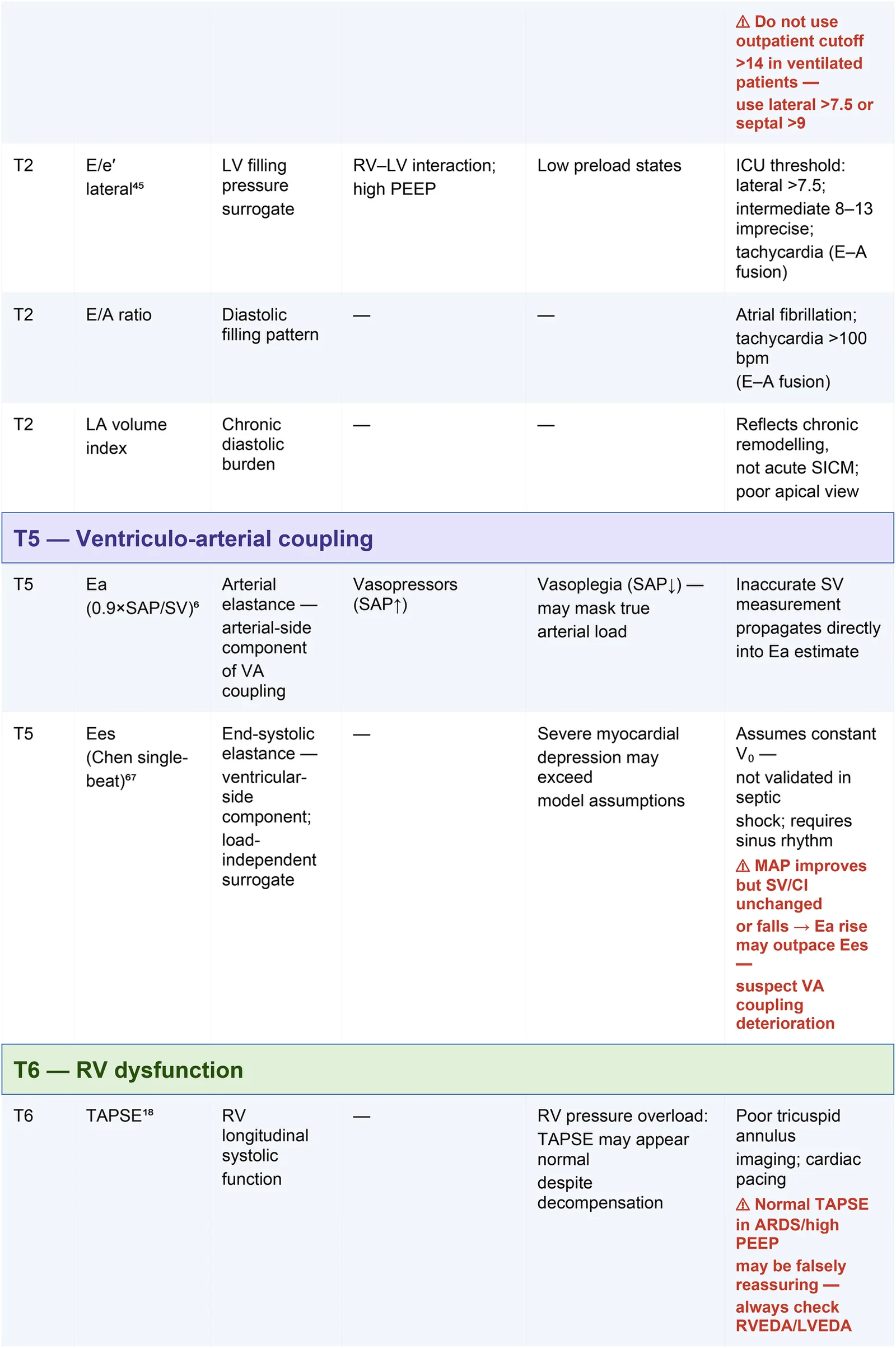


**Figure:**
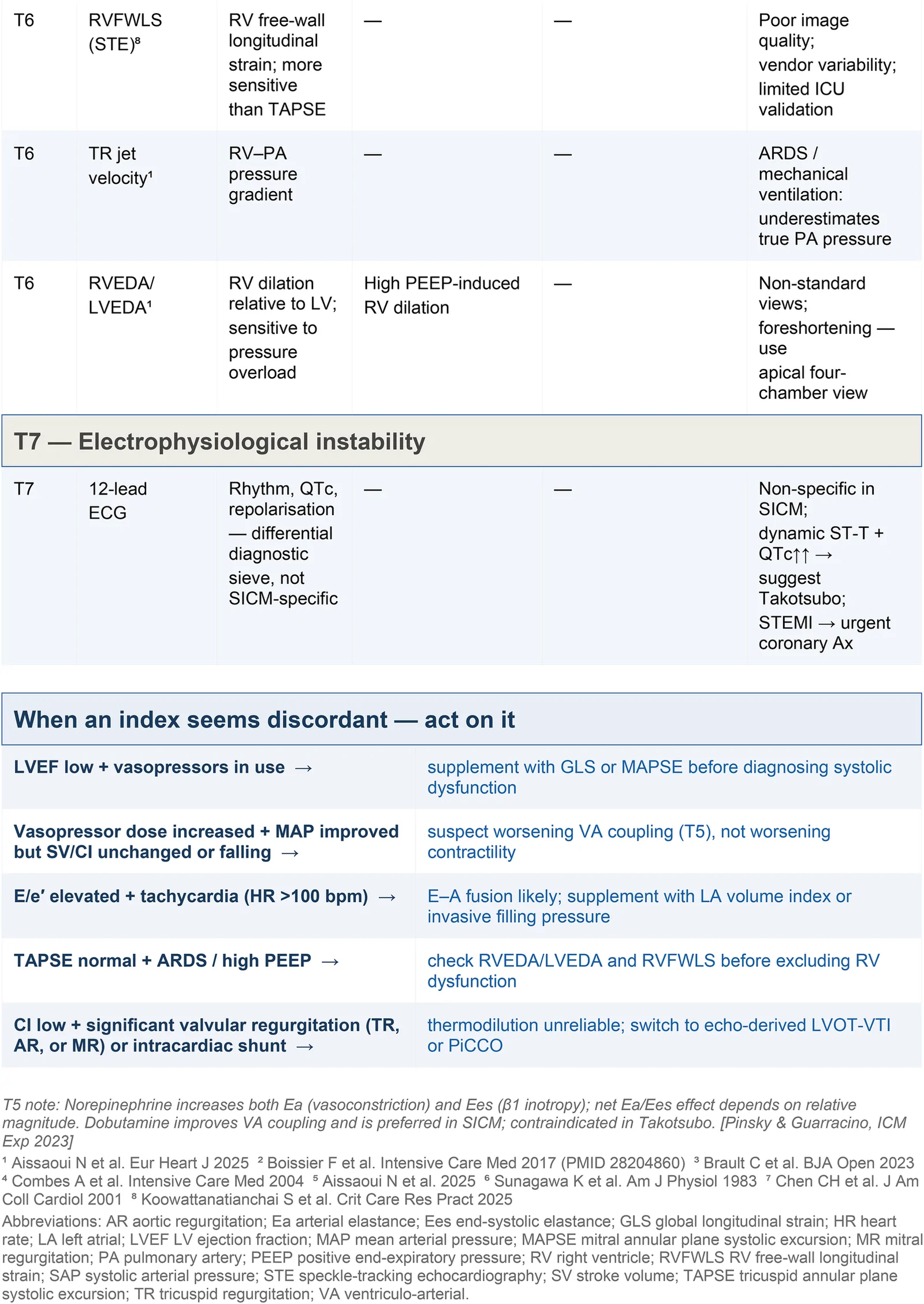


**Figure:**
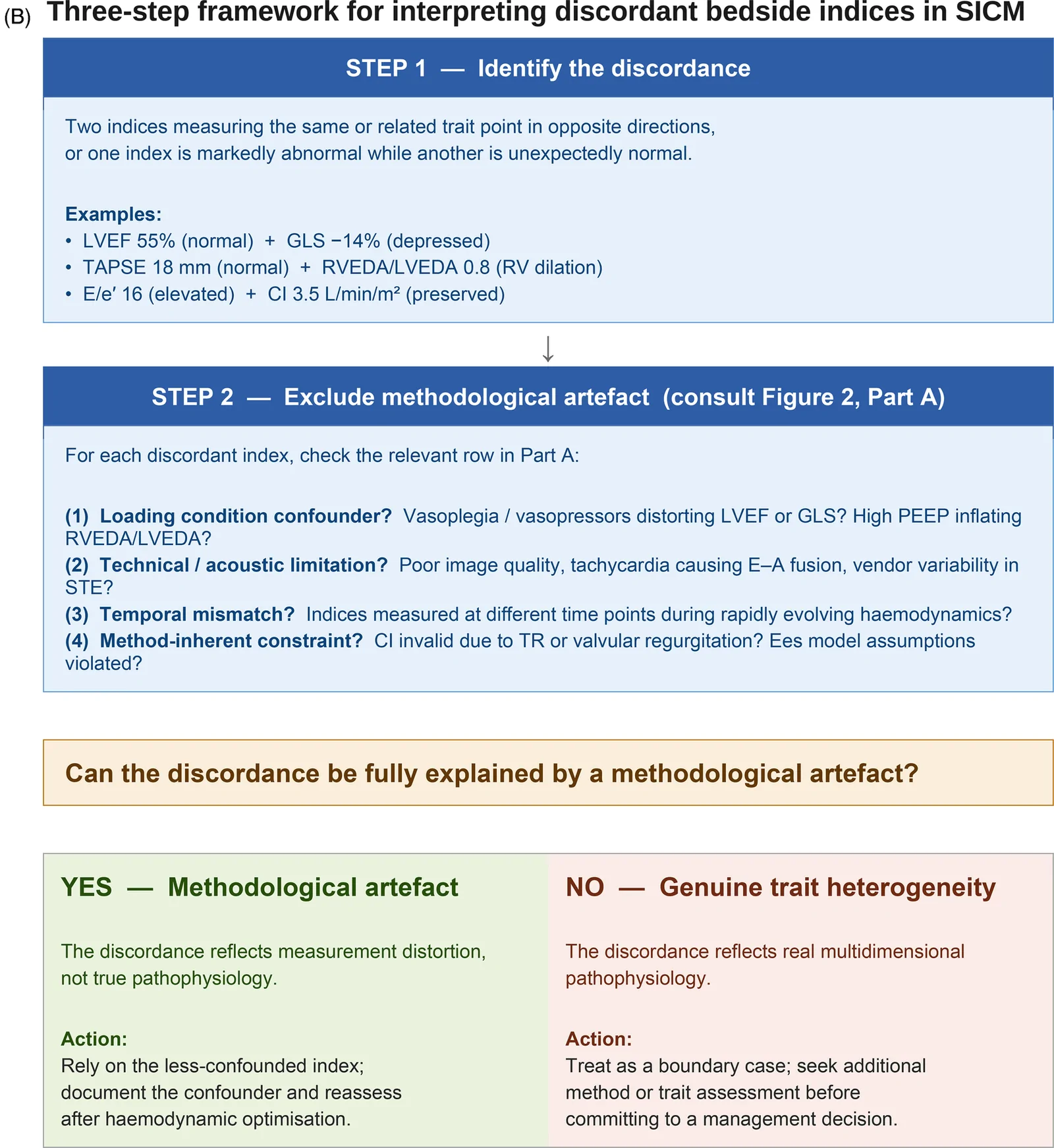


**Figure:**
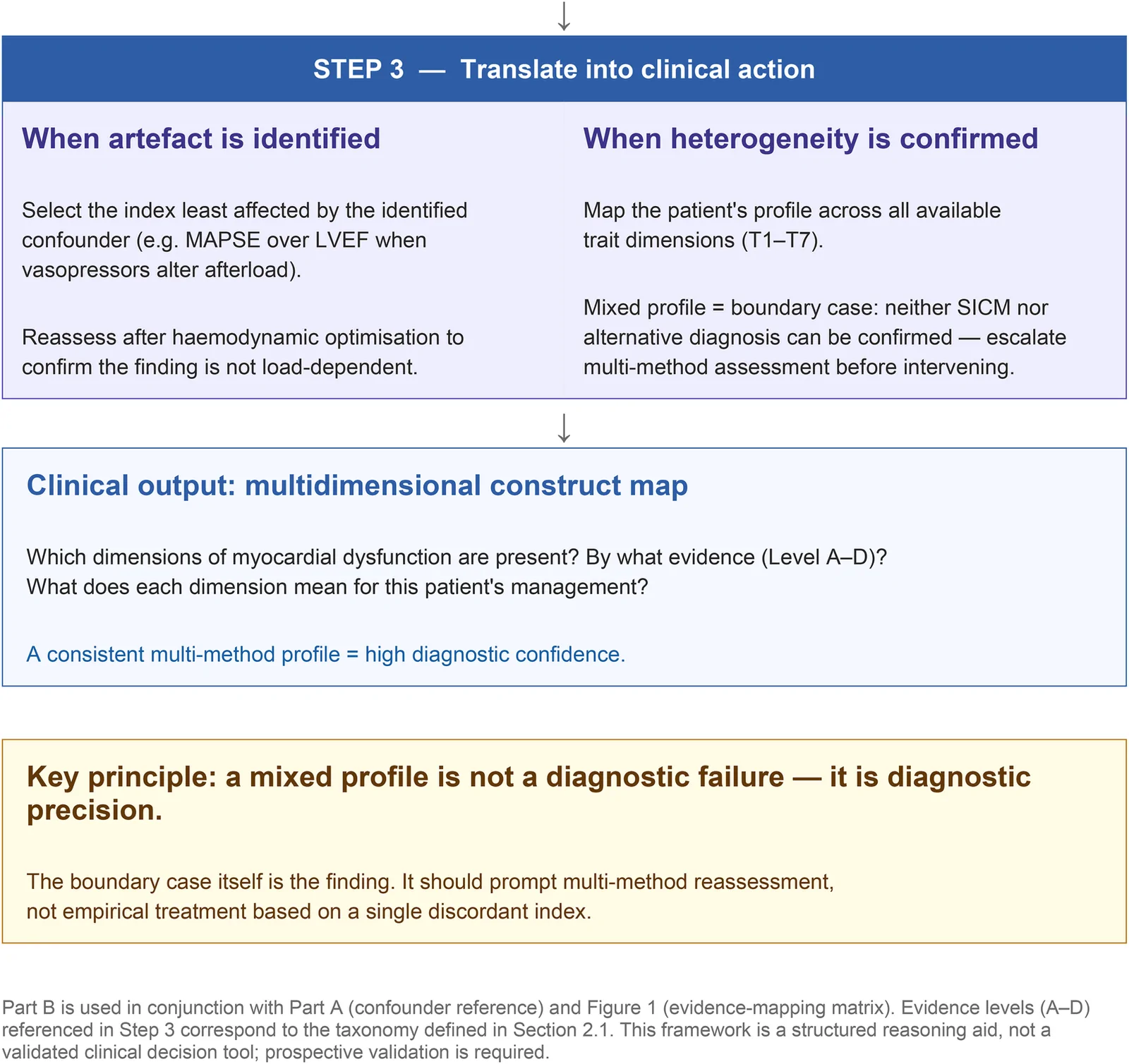


### 2.2 T1: systolic dysfunction

T1 achieves the strongest cross-method convergence in the framework: level A evidence across TTE, STE, and PAC/PiCCO (Figure 1). However, this convergence is conditional. LVEF is a volumetric ratio shaped as much by loading conditions as by intrinsic contractility; vasoplegia overestimates and vasopressor-driven afterload elevation underestimates (14, 15). MAPSE and GLS are less preload-sensitive but remain afterload-dependent: when E_a_ increases under vasopressor escalation, both fall even when intrinsic contractility is intact (14, 16). The diagnostic implication is that LVEF depression without corroborating MAPSE or GLS impairment does not satisfy the convergent validity criterion for T1; it more likely reflects T5 pathophysiology. Genuine T1 evidence requires that after preload optimisation and afterload contribution are excluded, impairment persists across at least two independent systolic indices. Specific loading-condition confounders for each T1 index are detailed in Figure 2A, along with a side-by-side comparison of the echocardiographic indices.

Troponin belongs to this dimension as an M4 index of myocyte injury rather than contractility (level B). It tracks diastolic dysfunction and right ventricular (RV) dilatation more closely than LVEF (17), discriminates echocardiographically defined cardiac dysfunction poorly [area under the curve (AUC), 0.65] (18), and does not correlate with cytokine levels, whereas N-terminal pro-B-type natriuretic peptide (NT-proBNP) does (19). Therefore, it is a non-specific injury signal that should prompt echocardiographic assessment and is not a marker of any single dimension.

### 2.3 T2: diastolic dysfunction

T2 achieves level A convergence across TTE and STE (20, 21), but it carries a mechanistic distinction from classical heart failure that standard echocardiographic grading cannot resolve. In SICM, cytokine-mediated SERCA2a suppression impairs active relaxation (22), while reduced myofilament calcium sensitivity increases the fibre length and myocardial oedema increases the wall water content, where both are proposed to contribute to increased passive compliance (23, 24)—the oedema link resting so far on a preprint—producing the defining haemodynamic signature of normal or low filling pressures despite systolic depression [pulmonary capillary wedge pressure (PCWP), 11–14 mmHg] (5, 25, 26). This compliance elevation buffers left atrial pressure, causing E/e′ to remain below standard diagnostic thresholds even when active relaxation is significantly impaired—the most common source of underdiagnosis of T2 in septic shock. In classical decompensated heart failure, reduced compliance drives filling pressure elevation and reliable E/e′ elevation; the same echocardiographic signal therefore implies opposite fluid management strategies in the two conditions. Troponin and NT-proBNP increase with diastolic dysfunction in septic cohorts and provide indirect (level B) support (17, 20). T2 diagnosis requires convergence across at least two independent signals—rate-controlled E/e′, left atrial enlargement, or directly measured filling pressures—interpreted alongside ventricular size; the tachycardia, positive end-expiratory pressure (PEEP), and volume-loading confounders affecting the E/e′ interpretation are detailed in Figure 2A (27).

### 2.4 T3: metabolic dysfunction

T3 is the structurally darkest region of the framework: all bedside methods return D and CMR only C, with circulating biomarkers as the sole level A source (Figure 1). This is not a temporary gap awaiting better technology; it reflects the absence of any routine bedside measurement of intracellular energetics. Magnetic resonance spectroscopy has not been applied in human sepsis, and T2-mapping of oedema in the one acute-phase CMR series (28) is a tissue surrogate two steps removed from energetics. The pathophysiology involves coordinated collapse of fatty acid oxidation, glucose oxidation, and tricarboxylic acid (TCA) cycle integrity, producing energetic paralysis in a structurally intact myocardium: an upstream failure of energy supply distinct from T1's downstream failure of the contractile apparatus (29). The trial design implication is unique: unlike T4, where construct dilution is the primary failure mode, T3 interventions face an additional problem—the therapeutic window for reversing mitochondrial injury may close before standard haemodynamic enrolment criteria are met. The most clinically accessible T3 proxies are plasma kynurenic acid and acetylcarnitine, which discriminated echocardiographically defined cardiac dysfunction (kynurenic acid AUC 0.80) in an 80-patient untargeted metabolomics study (18); long-chain acylcarnitines predict sepsis mortality (30, 31) but have not been related to cardiac dysfunction. None is in routine ICU use. Until validated early T3 biomarkers exist, this dimension will remain unreachable at enrolment—the most urgent unmet methodological need in the SICM trial design.

### 2.5 T4: inflammatory and immune dysregulation

T4 presents the framework's central paradox: level A evidence from circulating biomarkers (M4) yet the targets of any T4-directed intervention—myocardial NF-κB activation and cardiac-specific iNOS upregulation (32)—have no bedside measurement equivalent. Systemic inflammatory markers [IL-6, ferritin, and C-reactive protein (CRP)] reflect systemic inflammatory burden and are associated with SICM outcomes (13, 19), but they cannot confirm the presence or magnitude of cardiac-level inflammatory signalling; they are necessary but not sufficient surrogates for T4 cardiac targets. Corticosteroids act systemically and were never trialled as SICM-specific agents, and therefore, their net mortality risk ratio of 0.93 across 45 RCTs (9) cannot be read as a test of T4-directed therapy. Because cardiac T4 targets were never confirmed in enrolled patients, that literature is uninformative about whether cardiac inflammatory signalling is modifiable. Cardiac-specific microRNAs (miR-208a and miR-499) represent the most promising direction for future T4 cardiac-specific indicators, but they remain unvalidated.

### 2.6 T5: ventriculo-arterial coupling

T5 achieves level A evidence via TTE and PAC/PiCCO and level C via STE (Figure 1), and it provides the framework's most immediate inotrope-sparing clinical application. VA coupling deterioration can occur with E_es_ being entirely normal—driven solely by E_a_ elevation under vasopressor escalation—representing a system-level mismatch with no intrinsic myocardial pathology (33). Guarracino et al. found that 84% of patients with septic shock had E_a_/E_es_ >1.36 on ICU admission, confirming VA decoupling as a structural feature of septic shock rather than as an occasional finding (34). In subsequent transthoracic cohorts using the single-beat E_es_ estimate (35), baseline E_a_/E_es_ predicted the stroke-volume response to norepinephrine (36), E_a_/E_es_-guided resuscitation improved lactate clearance in a pilot randomised trial (37), and the change in E_a_/E_es_ during resuscitation was associated with 28-day mortality (38); the single-beat method has not been validated against invasive pressure-volume loops in sepsis. Speckle-tracking myocardial work has been reported in SICM only as a case series (39). The T1/T5 discriminating clue lies in the LVEF–MAPSE (or LVEF–GLS) relationship: the T1-dominant pathology produces proportionate impairment of both, and the T5-dominant pathology suppresses LVEF more than longitudinal shortening because MAPSE and GLS reflect intrinsic fibre behaviour more directly than the volumetric ratio. This LVEF–MAPSE discordance is the opening case's key signal. The functional vasopressor reduction test—cautious norepinephrine reduction while observing whether the stroke volume increases without proportionate pressure fall—follows from the E_a_/E_es_–norepinephrine relationship (36); how it is performed is described in Section 3.1. Dynamic arterial elastance (pulse pressure variation/stroke volume variation, PPV/SVV) and the specific coupling confounders are detailed in Figure 2A.

### 2.7 T6: right ventricular dysfunction

T6 achieves the broadest multi-method convergence in the framework after T1: level A across TTE, STE, and PAC/PiCCO, with level B from biomarkers (Figure 1). Its prognostic weight is at least equal to that of T1: RV dysfunction carried an approximately 2.4-fold increase in short-term mortality in a 1,373-patient meta-analysis (40), and in one 104-patient cohort, it had an adjusted 28-day hazard ratio of 5.53, while LVEF lost significance in the same model (41)—a cohort-specific estimate. That RV dysfunction can occur with normal LVEF has been known since five of 25 catheterised patients with septic shock showed exactly that (42), and RV strain now identifies it with high accuracy (43). These findings argue strongly against the left-heart-dominant monitoring approach that governs most ICU practice. The cross-dimensional clinical significance of T6 lies in the septal shift mechanism: RV dilation compresses the LV end-diastolic volume, producing LV underfilling with normal or elevated LVEF—a scenario in which the inotrope target (LV contractility) is not the problem, and the correct intervention is RV preload reduction through diuresis or ultrafiltration to release septal shift and restore LV filling (44). This is the physiological basis of reverse resuscitation (Section 3.1), and it is invisible within a left-heart-only monitoring framework. Tricuspid annular plane systolic excursion (TAPSE), the right-to-left ventricular end-diastolic area ratio (RVEDA/LVEDA), and the high PEEP confounders that can make TAPSE falsely reassuring are detailed in Figure 2A.

### 2.8 T7: electrophysiological instability

T7 occupies a unique position: level A evidence for ECG (45, 46) but discriminant validity functionally D (Figure 1). ECG neither establishes nor refutes SICM; its clinical value is exclusionary. New ST elevation mandates urgent myocardial infarction (MI) exclusion, new left bundle branch block (LBBB) alters the echocardiographic interpretive framework, and dynamic ST-T changes with QTc prolongation should raise a suspicion of Takotsubo syndrome. One cross-dimensional signal deserves explicit mention: new-onset atrial fibrillation (AF) in septic shock may be a clinical manifestation of T2, because elevated left atrial pressure and left atrial enlargement are recognised AF substrates and left atrial strain predicts new-onset AF in septic shock (47). Its occurrence should prompt simultaneous T2 assessment rather than isolated rhythm management.

## 3 Clinical translation

### 3.1 Three bedside questions

The framework does not replace clinical judgement. It structures it. Three questions recur when indices disagree at the bedside, each with a corresponding reasoning pathway.

Is this genuine systolic dysfunction or a loading artefact? When LVEF falls below the threshold but MAPSE (or GLS) is relatively preserved, the T1 convergent validity criterion is not met. Assess VA coupling before escalating inotropes: the functional vasopressor reduction test is the simplest bedside approach. If the stroke volume increases immediately without a proportionate fall in arterial pressure, the problem is afterload excess. Adding dobutamine to an afterload-uncoupled ventricle risks worsening the E_a_/E_es_ ratio, reducing diastolic filling time, and increasing myocardial oxygen demand—the opposite of the intended effect. With the mean arterial pressure at target and preload adequate, norepinephrine is reduced by 0.1–0.2 μg/kg/min; if pressure holds for 3–5 min, the dose is reduced again, and the step at which the pressure begins to fall is reversed—the same logic as a PEEP titration. The stroke volume must be visible during the process [pulse contour analysis or serial left ventricular outflow tract (LVOT) velocity-time integral], because the question is not whether pressure is maintained but whether the stroke volume increases as afterload is withdrawn.

Is diastolic dysfunction driving haemodynamic instability? In any patient with unexplained fluid intolerance, pulmonary oedema disproportionate to LVEF, or persistent hypotension despite adequate preload correction, T2 assessment is mandatory. The T2-dominant patient requires heart rate control, cautious diuresis when haemodynamics permit, and avoidance of tachycardia-provoking interventions—not further volume loading, which compounds filling pressure elevation without improving forward flow.

What does the full phenotype profile reveal? The framework provides a risk stratification signal independent of LVEF: a patient with T1 + T2 + T6 convergent evidence carries a categorically higher risk than one with isolated T1 evidence on a single method. The RV dimension carries independent prognostic weight that standard left-heart monitoring cannot capture (40, 41), and its identification directly changes the therapeutic target—from inotropes to reverse resuscitation. T3 and T4 do not enter this bedside profile: the metabolic and cardiac-specific inflammatory markers discussed in Sections 2.4 and 2.5 are a research and trial-enrolment agenda and not tools that can be ordered today—and the profile that a clinician can actually assemble is built from T1, T2, T5, T6, and T7. Reverse resuscitation is for the patient with RV dilation (RVEDA/LVEDA >0.6 with septal flattening) who is not fluid-responsive, after hypoxaemia, hypercapnia, acidosis, and excessive PEEP have been corrected. Fluid is removed stepwise against three references—central venous pressure, cardiac output, and lactate—and the diagnosis is confirmed by the response: central venous pressure falls, cardiac output rises rather than falls, and serum lactate does not increase. TAPSE alone is not sufficient to make the diagnosis (Figure 2A).

### 3.2 When SICM meets Takotsubo: discriminant validity in practice

The distinction between SICM and Takotsubo cardiomyopathy (TTS) tests the framework's discriminant validity and reveals what a single-method assessment cannot achieve. Both conditions produce reversible LV dysfunction, LVEF depression, and biomarker elevation; T1 converges at level A for both. The discriminating information lies in the T2 dimension.

In SICM, cytokine-mediated SERCA2a suppression impairs active relaxation, while compliance increases; the dilated ventricle buffers filling pressure, and PCWP remains normal or low (5, 25, 26). In TTS, the catecholamine surge drives β-AR coupling from G_s_ to G_i_ (stimulus trafficking) (48, 49), reducing protein kinase A (PKA) activity and impairing PKA-mediated phosphorylation of troponin I and myosin-binding protein C—the molecular basis of lusitropy. Active relaxation is prolonged and tau is extended, but passive compliance is preserved (OCTOPUS study) (50). The non-dilated ventricle cannot buffer delayed relaxation, and filling pressures may increase, particularly with tachycardia.

The wall motion pattern adds a second discriminating dimension: SICM produces global depression, while TTS produces the apical-basal gradient; MAPSE may be relatively preserved or elevated in TTS because basal function is spared, while GLS shows disproportionate impairment from apical involvement. This MAPSE-GLS dissociation is the echocardiographic fingerprint of TTS. The therapeutic implication is direct: dobutamine may benefit SICM where SERCA2a suppression underlies diastolic impairment, but it risks worsening the catecholaminergic injury in TTS. A trial that cannot distinguish these subgroups at enrolment cannot generate an interpretable result for either. The MAESTRO trial (NCT05793008) may provide the enrolment tools needed to resolve this boundary.

## 4 Why trials fail and what future trials need

We hypothesise that the therapeutic impasse is not only a failure of pharmacology but also a failure of construct validity in trial design, operating through two distinct mechanisms.

The first is construct dilution from T1-derived enrolment. Virtually every SICM trial has enrolled participants with depressed LVEF or haemodynamic instability—criteria that identify apparent systolic dysfunction and not specific pathophysiological processes. As the framework demonstrates, identical LVEF values emerge from mechanistically distinct sources; T1-based enrolment guarantees pathophysiological heterogeneity in the study population, diluting any treatment effect specific to one mechanism.

The second is structural target invisibility for T3 and T4 interventions. Any therapy directed at cardiac inflammatory signalling (T4) cannot have its target confirmed at the bedside; systemic markers are necessary but insufficient surrogates (9). Metabolic support targets intracellular energetic failure (T3), which has no bedside measurement equivalent and whose therapeutic window may close before the enrolment criteria are met. These are not problems that larger sample sizes alone can solve. The solution requires T3-specific early biomarkers [kynurenic acid and acetylcarnitine, pending prospective validation (18)] and T4 cardiac-specific indicators (cardiac microRNAs, pending validation) to enable mechanistically targeted enrolment—a research agenda that the framework makes explicit by indicating precisely where the current gaps are.

### 4.1 Limitations

This framework is conceptual and has not been prospectively validated. It is not a systematic review: the literature was identified by iterative search and hand searching without a registered protocol, preferred reporting items for systematic reviews and meta-analyses (PRISMA) reporting, or risk-of-bias assessment, and relevant studies may have been missed. Evidence grading (A–D) reflects our literature synthesis against the criteria in Section 2.1 and is inherently subjective in borderline cases, even with high inter-rater agreement; a formal Delphi-based international consensus would provide the external validation that this framework currently lacks. The selection of the seven dimensions is itself a judgement, and the multitrait-multimethod (MTMM) analogy is applied heuristically rather than formally (Section 2.1). Trait definitions are provisional: T3 and T4 are sufficiently entangled that a future iteration might merge them into a single metabolic inflammatory dimension. Several claims rest on limited evidence: the oedema compliance mechanism depends on a preprint (24), the T3 biomarker evidence depends on a single 80-patient study (18), and the T5 cohort data depends largely on one group (36–38). The framework addresses acute septic shock; its applicability to post-sepsis cardiomyopathy requires separate evaluation. The bedside reasoning framework is a structured aid and not a validated clinical decision tool, and the interventional pathways in Section 3.1 (functional vasopressor reduction and reverse resuscitation) are hypothesis-generating and potentially harmful if applied outside the conditions described there; prospective outcome studies are required before any of them can be recommended as a clinical protocol.

## 5 Conclusion

We return to the case of the 68-year-old man in septic shock. An LVEF of 40% with MAPSE at the lower limit of normal is not a convergent T1 signal; it is a discordant pattern consistent with E_a_/E_es_ deterioration in a myocardium whose intrinsic contractile capacity may be substantially better than what the LVEF suggests. Before escalating inotropes, the framework directs the clinician to test VA coupling: once preload deficit is excluded and stroke volume monitored, norepinephrine is reduced cautiously while observing whether the stroke volume increases. If it does, the problem is afterload excess, and it is possible that dobutamine would have worsened it.

This is what the framework offers: not a protocol but a structured way to ask better questions. The discordance between LVEF and MAPSE is not a problem to be resolved by choosing the better index. It is information—about which dimensions of cardiac function are and are not affected in this patient at this moment. SICM will not have a gold standard in the near term. The bedside clinician cannot wait for one. The framework does not resolve the underlying heterogeneity of SICM; it makes that heterogeneity legible. Whether legibility changes outcomes is a testable question, and the three-step algorithm should now be tested in prospective cohorts: patients profiled at the bedside across the five measurable dimensions, managed according to the profile, and followed for the haemodynamic response that confirms or refutes each phenotype. Such a study can be done with the tools already available in every ICU.
