# Supplementary material for: Reading discordant bedside indices in sepsis-induced cardiomyopathy: an evidence-mapping framework for diagnosis and trial design
Source: Front Cardiovasc Med. 2026 Sep 29;13:1918976. doi: 10.3389/fcvm.2026.1918976 (PMC13658951; doi:10.3389/fcvm.2026.1918976)
Supplement: Supplementary file 1 [file Datasheet1.docx]

# **Supplementary Table S1. Cell-by-cell grading of the evidence-mapping matrix (Figure 1)**

*This table documents the grade assigned to each of the 42 trait-method cells in Figure 1, the criterion on which the grade rests, and the key references supporting it. It is intended to allow readers to audit every cell independently. Full citations are given in Section 4.*

## **1. Grading criteria**

Grade A — direct evidence. At least one clinical study in adult sepsis or septic shock in which the method measured an index of the trait and reported (i) prevalence or diagnostic performance against another method, or (ii) an association with a clinical outcome. Reviews alone do not satisfy criterion A.

Grade B — indirect inference. The method measures an index that maps onto the trait, but the evidence is (i) from sepsis at a different phase from that specified in the cell, (ii) from non-sepsis populations or animal sepsis models, or (iii) from sepsis studies in which the index is a surrogate whose validity for the trait has not been established in sepsis (for example, natriuretic peptides as an index of filling pressure).

Grade C — theoretical link. A measurement pathway is proposed in mechanistic or review literature, but no cohort measurement of the trait by this method has been reported in sepsis, or a state-of-the-art review explicitly states that clinical data are lacking. Isolated case reports do not raise a cell above C.

Grade D — blank. D-conceptual: the method cannot measure the trait in principle (for example, ECG and contractility). D-empirical: measurement is technically conceivable but no data exist in sepsis.

Time stratification (t). Where evidence differs between the acute phase (<48 h) and the recovery phase (>7–14 d), the grade is given for the phase in which evidence exists. Acute-phase CMR evidence derives from a single series of highly selected patients who could be transported to the scanner and does not reflect routine feasibility.

## **2. Independent grading and agreement**

Both authors (L.Z. and S.Z.) independently graded all 42 cells against the criteria in Section 1, without reference to each other’s grades. Agreement was reached in 41 of 42 cells (observed agreement 97.6%; Cohen’s κ = 0.96). The single discordant cell, T3×M3 (metabolic dysfunction × CMR), was graded B by one author and C by the other; it was resolved by consensus in favour of C, because the only CMR finding in acute septic shock that has been proposed as a correlate of metabolic stress is T2-mapping oedema, which is a tissue surrogate rather than a measure of energetics, and magnetic resonance spectroscopy has not been applied in human sepsis. The final distribution is 13 cells graded A, 7 graded B, 2 graded C and 20 graded D.

## **3. Cell-by-cell table**

| **Cell** | **Trait × Method** | **Grade** | **Basis for grade (criteria in Section 1)** | **Key references** |
| --- | --- | --- | --- | --- |
| **T1×M1** | Systolic dysfunction × TTE | A | Criterion A. LVEF, MAPSE and tissue-Doppler s′ have been measured by TTE in multiple septic-shock cohorts, with prevalence estimates, characterisation of loading-condition dependence, and outcome associations (including the U-shaped LVEF–mortality relationship). | Jardin 1990; De Geer 2015; Boissier 2017; Brault 2023; Dugar 2023; Hasegawa 2023; Aissaoui 2025 |
| **T1×M2** | Systolic dysfunction × STE | A | Criterion A. Global longitudinal strain has been measured by STE in septic-shock cohorts, correlates with LVEF and NT-proBNP, detects subclinical dysfunction, and is associated with mortality in meta-analyses. Note that STE requires offline post-processing and is therefore a near-bedside rather than bedside method. | De Geer 2015; Boissier 2017; Sanfilippo 2018; Pruszczyk 2024 |
| **T1×M3** | Systolic dysfunction × CMR | B (t) | Criterion B. CMR has quantified LV function in one acute-phase series of critically ill septic-shock patients who could be transported, and in sepsis survivors weeks after the episode. Evidence is indirect because the acute series is highly selected and the recovery series measures a different trait state; CMR is not feasible in most vasopressor-dependent patients. | Muehlberg 2022; Jacobs 2025; Malomo 2025; Leo 2025 |
| **T1×M4** | Systolic dysfunction × Biomarkers | B | Criterion B. Troponin and natriuretic peptides are routinely measured in sepsis but index myocyte injury and wall stress rather than contractility; troponin I (AUC 0.65) and NT-proBNP (AUC 0.63) discriminate echocardiographically defined cardiac dysfunction poorly, and troponin is uncorrelated with cytokine levels. They are surrogates not validated as T1 measures. | Landesberg 2014; Cao 2023; Nedel 2026; Guarino 2025 |
| **T1×M5** | Systolic dysfunction × PAC/PiCCO | A | Criterion A. Cardiac index and stroke-work indices were measured by PAC in the original descriptions of septic myocardial depression, and PAC-guided management has been studied in sepsis-associated cardiogenic shock. Cardiac index is a composite of T1, T5 and T6 (see Figure 2A). | Parker 1984; Jardin 1990; Sato 2024 |
| **T1×M6** | Systolic dysfunction × ECG | D-conceptual | The ECG carries no measure of contractile performance; the trait is not measurable by this method in principle. | — |
| **T2×M1** | Diastolic dysfunction × TTE | A | Criterion A. E/e′, E/A and tissue-Doppler e′ have been measured in septic-shock cohorts, validated against pulmonary artery occlusion pressure, and associated with mortality in cohort and meta-analytic data; prevalence is 48–76%. | Combes 2004; Bouhemad 2008; Landesberg 2012; Sanfilippo 2015; Sanfilippo 2017; Vignon 2025 |
| **T2×M2** | Diastolic dysfunction × STE | A | Criterion A. A prospective septic-shock cohort (n = 98) applied STE-based diastolic criteria serially on days 1, 7 and 30, identifying diastolic dysfunction in 52% versus 51% by conventional criteria; STE-derived left atrial strain measured in septic shock predicts new-onset atrial fibrillation. Left atrial reservoir strain is validated against invasive filling pressure outside sepsis. | Gonzalez 2025; Beyls 2024; Gonzalez 2024 |
| **T2×M3** | Diastolic dysfunction × CMR | B (t, acute) | Criterion B. T2-mapping myocardial oedema in the acute-phase septic-shock CMR series is a plausible tissue correlate of the compliance change underlying T2, but oedema is a tissue surrogate rather than a diastolic-function measure, and the series is highly selected. | Muehlberg 2022; Jacobs 2025 |
| **T2×M4** | Diastolic dysfunction × Biomarkers | B | Criterion B. In a 262-patient septic cohort, echocardiographic diastolic dysfunction was accompanied by significantly higher high-sensitivity troponin T and NT-proBNP; troponin elevation in sepsis is associated with diastolic dysfunction and RV dilatation; NT-proBNP correlates with vasopressor load. Biomarkers index wall stress and injury rather than relaxation, so evidence is indirect. | Landesberg 2012; Landesberg 2014; Nedel 2026 |
| **T2×M5** | Diastolic dysfunction × PAC/PiCCO | B | Criterion B. Pulmonary artery occlusion pressure is directly measured in septic-shock cohorts, but it reflects filling pressure rather than relaxation or compliance and is buffered by the increased ventricular compliance characteristic of SICM; it is therefore an indirect index of T2. | Parker 1984; Jardin 1990; Combes 2004 |
| **T2×M6** | Diastolic dysfunction × ECG | D-conceptual | The ECG cannot measure relaxation or filling pressure. New-onset atrial fibrillation is documented in sepsis and is associated with left atrial strain, which supports the hypothesis in Section 2.8 that AF may be a downstream manifestation of T2, but does not make the ECG a T2 measurement. | Walkey 2011; Beyls 2024 |
| **T3×M1** | Metabolic dysfunction × TTE | D-conceptual | Echocardiography measures mechanical output, not intracellular energetics; 95% of myocardial ATP derives from mitochondrial oxidative phosphorylation, which has no acoustic correlate. | Yu 2025 |
| **T3×M2** | Metabolic dysfunction × STE | D-conceptual | As T3×M1. | Yu 2025 |
| **T3×M3** | Metabolic dysfunction × CMR | C | Criterion C. 31P-magnetic resonance spectroscopy can quantify myocardial phosphocreatine/ATP, but it has not been applied in human sepsis; the only CMR finding in acute septic shock that has been proposed as a correlate of metabolic stress is T2-mapping oedema, which is a tissue surrogate two steps removed from energetics. The link is theoretical. | Muehlberg 2022; Leo 2025 |
| **T3×M4** | Metabolic dysfunction × Biomarkers | A | Criterion A. Untargeted plasma metabolomics in 80 septic patients (43 with echocardiographically defined cardiac dysfunction) identified kynurenic acid as an independent discriminator of cardiac dysfunction (AUC 0.80) and of 28-day mortality within the dysfunction group, with acetylcarnitine also elevated. In broader sepsis cohorts, acylcarnitines and acetylcarnitine are associated with mortality and organ dysfunction; long-chain acylcarnitines have not yet been related to cardiac dysfunction in sepsis. | Cao 2023; Langley 2013; Chung 2019; Guarino 2025; Yu 2025 |
| **T3×M5** | Metabolic dysfunction × PAC/PiCCO | D-conceptual | Haemodynamic monitoring quantifies flow and pressure, not substrate utilisation; not measurable in principle. | — |
| **T3×M6** | Metabolic dysfunction × ECG | D-conceptual | As T3×M5. | — |
| **T4×M1** | Inflammation/immunity × TTE | D-conceptual | Echocardiography cannot resolve cardiac inflammatory signalling; the myocardial transcriptomic and proteomic signature of sepsis has no echocardiographic correlate. | — |
| **T4×M2** | Inflammation/immunity × STE | D-conceptual | As T4×M1. | — |
| **T4×M3** | Inflammation/immunity × CMR | D-empirical | T2 mapping and late gadolinium enhancement could in principle index myocardial inflammation, as they do in myocarditis, but no sepsis CMR study has related these findings to inflammatory markers, and recovery-phase fibrosis does not discriminate an inflammatory mechanism. | Muehlberg 2022; Jacobs 2025 |
| **T4×M4** | Inflammation/immunity × Biomarkers | A† | Criterion A for systemic markers. IL-6, IL-10, CRP and ferritin are measured in septic-shock cohorts and associated with SICM outcomes; NT-proBNP correlates with IL-6 and IL-10 whereas troponin does not. †Cardiac-specific NF-κB and iNOS signalling has no bedside equivalent, so systemic markers are necessary but insufficient surrogates. | Finkel 1992; Flierl 2008; Martin 2019; Nedel 2026; Guarino 2025 |
| **T4×M5** | Inflammation/immunity × PAC/PiCCO | D-conceptual | Not measurable in principle. | — |
| **T4×M6** | Inflammation/immunity × ECG | D-conceptual | Not measurable in principle. | — |
| **T5×M1** | Ventriculo-arterial coupling × TTE | A | Criterion A. TTE-derived Ea/Ees (Ea = 0.9 × SAP/SV; Ees by the single-beat method) has been measured in septic-shock cohorts: 84% were decoupled on admission (n = 25); baseline Ea/Ees predicted stroke-volume response to norepinephrine (n = 34); an Ea/Ees-guided resuscitation algorithm improved lactate clearance in a pilot randomised trial (n = 83); and the change in Ea/Ees during resuscitation was independently associated with 28-day mortality. Footnote: the single-beat method assumes a constant V0 and has not been validated against invasive pressure–volume loops in sepsis. | Chen 2001; Guarracino 2014; Zhou 2021; Zhou 2022; Zhou 2024; Pinsky 2023 |
| **T5×M2** | Ventriculo-arterial coupling × STE | C | Criterion C. The only STE-derived coupling-related index, non-invasive myocardial work from pressure–strain loops (GLS combined with arterial pressure), has been reported in SICM only as a three-patient case series; no cohort data exist, and a 2025 state-of-the-art review states that clinical data on ventriculo-arterial coupling in sepsis remain limited. | Salinas 2025; Sato 2025 |
| **T5×M3** | Ventriculo-arterial coupling × CMR | D-empirical | CMR-derived pressure–volume estimation is possible in stable patients but has never been applied in sepsis. | Leo 2025 |
| **T5×M4** | Ventriculo-arterial coupling × Biomarkers | D-conceptual | No biomarker indexes the Ea/Ees relationship. | — |
| **T5×M5** | Ventriculo-arterial coupling × PAC/PiCCO | A | Criterion A. Ea was derived from indwelling arterial-catheter pressure and PAC stroke volume in the same septic-shock cohort in which 21 of 25 patients had Ea/Ees > 1.36 on admission. Dynamic arterial elastance (PPV/SVV) from PiCCO is a further arterial-side surrogate. | Guarracino 2014; Pinsky 2023 |
| **T5×M6** | Ventriculo-arterial coupling × ECG | D-conceptual | Not measurable in principle. | — |
| **T6×M1** | RV dysfunction × TTE | A | Criterion A. TAPSE, tricuspid annular s′, fractional area change and RVEDA/LVEDA have been measured in septic-shock cohorts with prevalence (approximately 20–35%) and mortality associations: pooled approximately 2.4-fold risk in meta-analysis, an adjusted hazard ratio of 5.53 in a single 104-patient cohort, and a graded risk with the number of abnormal RV indices. | Vieillard-Baron 2020; Vallabhajosyula 2021; Geri 2019; Lu 2024; Koowattanatianchai 2025; Sato 2025; Aissaoui 2025 |
| **T6×M2** | RV dysfunction × STE | A | Criterion A. RV free-wall longitudinal strain and tissue motion annular displacement were measured by STE in a 159-patient sepsis cohort with diagnostic AUROC above 0.9 for RV dysfunction and univariable association with mortality; a prospective septic-shock cohort applied STE-based RV criteria serially. STE parameters were not independent predictors after multivariable adjustment and are therefore diagnostic rather than prognostic tools. | Yao 2025; Gonzalez 2025 |
| **T6×M3** | RV dysfunction × CMR | B (t, acute) | Criterion B. RV volumes and function were quantified in the acute-phase septic-shock CMR series in highly selected patients; survivor CMR found no RV late gadolinium enhancement. Evidence is indirect because of selection and because RV CMR indices have not been validated against outcome in sepsis. | Muehlberg 2022; Jacobs 2025 |
| **T6×M4** | RV dysfunction × Biomarkers | B | Criterion B. In a septic cohort, troponin elevation was associated with echocardiographic RV dilatation, and NT-proBNP correlates with vasopressor load; neither marker is specific for the right ventricle or can localise dysfunction. | Landesberg 2014; Nedel 2026 |
| **T6×M5** | RV dysfunction × PAC/PiCCO | A | Criterion A. Thermodilution RV ejection-fraction catheters documented RV dysfunction in septic shock (RVEF 23.8% versus 32.5% in controls; falling during shock and recovering with resolution), and a combined right-heart catheterisation and radionuclide study found depressed RVEF in 13 of 25 septic-shock patients, five of whom had normal LVEF. | Kimchi 1984; Vincent 1989; Sato 2024 |
| **T6×M6** | RV dysfunction × ECG | D-empirical | An RV strain pattern is non-specific and has not been evaluated against echocardiographic RV dysfunction in sepsis. | — |
| **T7×M1** | Electrophysiological instability × TTE | D-conceptual | Imaging does not characterise rhythm or repolarisation. | — |
| **T7×M2** | Electrophysiological instability × STE | D-conceptual | As T7×M1. | — |
| **T7×M3** | Electrophysiological instability × CMR | D-conceptual | As T7×M1. | — |
| **T7×M4** | Electrophysiological instability × Biomarkers | D-conceptual | No biomarker indexes electrophysiological instability. | — |
| **T7×M5** | Electrophysiological instability × PAC/PiCCO | D-conceptual | As T7×M4. | — |
| **T7×M6** | Electrophysiological instability × ECG | A† | Criterion A. New-onset atrial fibrillation and new-onset QT prolongation are documented in sepsis cohorts with outcome associations, and atrial strain predicts new-onset AF in septic shock. †The ECG neither establishes nor refutes SICM; its discriminant validity is functionally D and it serves as an exclusionary sieve. | Walkey 2011; Liu 2024; Beyls 2024 |

*Colour key: green, A; amber, B; red, C; grey, D. †T4×M4 and T7×M6: see the Basis column for the qualification attached to these grades.*

## **4. References cited in this table**

1. Aissaoui N, Boissier F, Chew M, Singer M, Vignon P. Sepsis-induced cardiomyopathy. Eur Heart J. 2025;46:3339–53. doi:10.1093/eurheartj/ehaf340

2. Beyls C, Hermida A, Daumin C, et al. Left and right atrial strain analysis to predicting new-onset atrial fibrillation in patients with septic shock: a single-center retrospective echocardiography study. Crit Care. 2024;28:233. doi:10.1186/s13054-024-05024-9

3. Boissier F, Razazi K, Seemann A, et al. Left ventricular systolic dysfunction during septic shock: the role of loading conditions. Intensive Care Med. 2017;43:633–42. doi:10.1007/s00134-017-4698-z

4. Bouhemad B, Nicolas-Robin A, Arbelot C, Arthaud M, Féger F, Rouby JJ. Isolated and reversible impairment of ventricular relaxation in patients with septic shock. Crit Care Med. 2008;36:766–74. doi:10.1097/CCM.0B013E31816596BC

5. Brault C, Zerbib Y, Mercado P, et al. Mitral annular plane systolic excursion for assessing left ventricular systolic dysfunction in patients with septic shock. BJA Open. 2023;7:100220. doi:10.1016/j.bjao.2023.100220

6. Cao Y, Liu Z, Ma W, et al. Untargeted metabolomic profiling of sepsis-induced cardiac dysfunction. Front Endocrinol (Lausanne). 2023;14:1060470. doi:10.3389/fendo.2023.1060470

7. Chen CH, Fetics B, Nevo E, et al. Noninvasive single-beat determination of left ventricular end-systolic elastance in humans. J Am Coll Cardiol. 2001;38:2028–34. doi:10.1016/S0735-1097(01)01651-4

8. Chung KP, Chen GY, Chuang TY, et al. Increased plasma acetylcarnitine in sepsis is associated with multiple organ dysfunction and mortality: a multicenter cohort study. Crit Care Med. 2019;47:210–8. doi:10.1097/CCM.0000000000003517

9. Combes A, Arnoult F, Trouillet JL. Tissue Doppler imaging estimation of pulmonary artery occlusion pressure in ICU patients. Intensive Care Med. 2004;30:75–81. doi:10.1007/s00134-003-2039-x

10. De Geer L, Engvall J, Oscarsson A. Strain echocardiography in septic shock – a comparison with systolic and diastolic function parameters, cardiac biomarkers and outcome. Crit Care. 2015;19:122. doi:10.1186/s13054-015-0857-1

11. Dugar S, Choudhary C, Duggal A. The U-shaped relationship between left ventricular ejection fraction and in-hospital mortality in patients with sepsis and septic shock. Chest. 2023;163:1414–24. doi:10.1016/j.chest.2023.01.010

12. Finkel MS, Oddis CV, Jacob TD, Watkins SC, Hattler BG, Simmons RL. Negative inotropic effects of cytokines on the heart mediated by nitric oxide. Science. 1992;257:387–9. doi:10.1126/science.1631560

13. Flierl MA, Rittirsch D, Huber-Lang MS, Sarma JV, Ward PA. Molecular events in the cardiomyopathy of sepsis. Mol Med. 2008;14:327–36. doi:10.2119/2007-00130.Flierl

14. Geri G, Vignon P, Aubry A, et al. Cardiovascular clusters in septic shock combining clinical and echocardiographic parameters: a post hoc analysis. Intensive Care Med. 2019;45:657–67. doi:10.1007/s00134-019-05597-2

15. Gonzalez FA, Santonocito C, Maybauer MO, Lopes LR, Almeida AG, Sanfilippo F. Diastology in the intensive care unit: challenges for the assessment and future directions. Echocardiography. 2024;41:e15773. doi:10.1111/echo.15773

16. Gonzalez FA, Bacariza J, Varudo AR, et al. Sepsis-induced myocardial dysfunction diagnosed with strain versus non-strain echocardiography parameters: incidence, evolution and association with prognosis. Ann Intensive Care. 2025;15:141. doi:10.1186/s13613-025-01561-w

17. Guarino M, Luppi F, Maroncelli G, et al. From cardiac injury to omics signatures: a narrative review on biomarkers in septic cardiomyopathy. Clin Exp Med. 2025;25:298. doi:10.1007/s10238-025-01842-5

18. Guarracino F, Ferro B, Morelli A, Bertini P, Baldassarri R, Pinsky MR. Ventriculoarterial decoupling in human septic shock. Crit Care. 2014;18:R80. doi:10.1186/cc13842

19. Hasegawa D, Ishisaka Y, Maeda T, et al. Prevalence and prognosis of sepsis-induced cardiomyopathy: a systematic review and meta-analysis. J Intensive Care Med. 2023;38:797–808. doi:10.1177/08850666231180526

20. Jacobs E, Malomo S, Oswald T, et al. Assessment of diffuse myocardial fibrosis and myocardial oedema in sepsis survivors using cardiovascular magnetic resonance: correlation with left ventricular systolic function. Biomedicines. 2025;13:2119. doi:10.3390/biomedicines13092119

21. Jardin F, Brun-Ney D, Auvert B, Beauchet A, Bourdarias JP. Sepsis-related cardiogenic shock. Crit Care Med. 1990;18:1055–60. doi:10.1097/00003246-199010000-00001

22. Kimchi A, Ellrodt AG, Berman DS, Riedinger MS, Swan HJ, Murata GH. Right ventricular performance in septic shock: a combined radionuclide and hemodynamic study. J Am Coll Cardiol. 1984;4:945–51. doi:10.1016/s0735-1097(84)80055-8

23. Koowattanatianchai S, Kochaiyapatana P, Eungsuwat N, et al. Significance of right ventricular dysfunction in predicting short-term survival among patients with sepsis and septic shock. Crit Care Res Pract. 2025;2025:5511135. doi:10.1155/ccrp/5511135

24. Landesberg G, Gilon D, Meroz Y, et al. Diastolic dysfunction and mortality in severe sepsis and septic shock. Eur Heart J. 2012;33:895–903. doi:10.1093/eurheartj/ehr351

25. Landesberg G, Jaffe AS, Gilon D, et al. Troponin elevation in severe sepsis and septic shock: the role of left ventricular diastolic dysfunction and right ventricular dilatation. Crit Care Med. 2014;42:790–800. doi:10.1097/CCM.0000000000000107

26. Langley RJ, Tsalik EL, van Velkinburgh JC, et al. An integrated clinico-metabolomic model improves prediction of death in sepsis. Sci Transl Med. 2013;5:195ra95. doi:10.1126/scitranslmed.3005893

27. Leo I, et al. Feasibility and role of cardiac magnetic resonance in intensive and acute cardiovascular care. J Clin Med. 2025;14:1112. doi:10.3390/jcm14041112

28. Liu W, Shao R, Zhang S, et al. Characteristics, predictors and outcomes of new-onset QT prolongation in sepsis: a multicenter retrospective study. Crit Care. 2024;28:115. doi:10.1186/s13054-024-04879-2

29. Lu NF, Pan L, Chen J, Chen X, Chen H. Types of septic cardiomyopathy: prognosis and influencing factors – a clinical study. Risk Manag Healthc Policy. 2024;17:1299–309. doi:10.2147/RMHP.S450933

30. Malomo S, Oswald T, Stephenson E, et al. Characterisation of post-sepsis cardiomyopathy using cardiovascular magnetic resonance. Diagnostics (Basel). 2025;15:997. doi:10.3390/diagnostics15080997

31. Martin L, Derwall M, Al Zoubi S, et al. The septic heart: current understanding of molecular mechanisms and clinical implications. Chest. 2019;155:427–37. doi:10.1016/j.chest.2018.08.1037

32. Muehlberg F, Blaszczyk E, Will K, et al. Characterization of critically ill patients with septic shock and sepsis-associated cardiomyopathy using cardiovascular MRI. ESC Heart Fail. 2022;9:2147–56. doi:10.1002/ehf2.13938

33. Nedel W, Portela LV. Association between troponin and NT-proBNP levels, cytokines, and clinical outcomes in early sepsis response: a cohort study. Braz J Anesthesiol. 2026;76:844712. doi:10.1016/j.bjane.2025.844712

34. Parker MM, Shelhamer JH, Bacharach SL, et al. Profound but reversible myocardial depression in patients with septic shock. Ann Intern Med. 1984;100:483–90. doi:10.7326/0003-4819-100-4-483

35. Pinsky MR, Guarracino F. Pathophysiological implications of ventriculoarterial coupling in septic shock. Intensive Care Med Exp. 2023;11:87. doi:10.1186/s40635-023-00573-9

36. Pruszczyk A, Zawadka M, Andruszkiewicz P, et al. Mortality in patients with septic cardiomyopathy identified by longitudinal strain by speckle tracking echocardiography: an updated systematic review and meta-analysis with trial sequential analysis. Anaesth Crit Care Pain Med. 2024;43:101339. doi:10.1016/j.accpm.2023.101339

37. Salinas PD, Barnett J, Dugar S. Sepsis and the heart: in the quest for noninvasive pressure-volume loops at the bedside. Crit Care Explor. 2025;7:e1328. doi:10.1097/CCE.0000000000001328

38. Sanfilippo F, Corredor C, Fletcher N, et al. Diastolic dysfunction and mortality in septic patients: a systematic review and meta-analysis. Intensive Care Med. 2015;41:1004–13. doi:10.1007/s00134-015-3748-7

39. Sanfilippo F, Corredor C, Arcadipane A, et al. Tissue Doppler assessment of diastolic function and relationship with mortality in critically ill septic patients: a systematic review and meta-analysis. Br J Anaesth. 2017;119:583–94. doi:10.1093/bja/aex254

40. Sanfilippo F, Corredor C, Fletcher N, et al. Left ventricular systolic function evaluated by strain echocardiography and relationship with mortality in patients with severe sepsis or septic shock: a systematic review and meta-analysis. Crit Care. 2018;22:183. doi:10.1186/s13054-018-2113-y

41. Sato R, Hasegawa D, Guo SC, Nishida K, Dugar S. Invasive hemodynamic monitoring with pulmonary artery catheter in sepsis-associated cardiogenic shock. Shock. 2024;61:712–7. doi:10.1097/SHK.0000000000002290

42. Sato R, Sanfilippo F, Lanspa M, Duggal A, Dugar S. Sepsis-induced cardiomyopathy: mechanism, prevalence, assessment, prognosis, and management. Chest. 2025;168:1383–94. doi:10.1016/j.chest.2025.08.013

43. Vallabhajosyula S, Shankar A, Vojjini R, et al. Impact of right ventricular dysfunction on short-term and long-term mortality in sepsis: a meta-analysis of 1,373 patients. Chest. 2021;159:2254–63. doi:10.1016/j.chest.2020.12.016

44. Vieillard-Baron A, Prigent A, Repessé X, et al. Right ventricular failure in septic shock: characterization, incidence and impact on fluid responsiveness. Crit Care. 2020;24:630. doi:10.1186/s13054-020-03347-1

45. Vignon P, Charron C, Legras A, et al. Left ventricular diastolic dysfunction is prevalent but not associated with mortality in patients with septic shock. Intensive Care Med. 2025;51:94–105. doi:10.1007/s00134-024-07748-2

46. Vincent JL, Reuse C, Frank N, Contempré B, Kahn RJ. Right ventricular dysfunction in septic shock: assessment by measurements of right ventricular ejection fraction using the thermodilution technique. Acta Anaesthesiol Scand. 1989;33:34–8.

47. Walkey AJ, Wiener RS, Ghobrial JM, Curtis LH, Benjamin EJ. Incident stroke and mortality associated with new-onset atrial fibrillation in patients hospitalized with severe sepsis. JAMA. 2011;306:2248–54. doi:10.1001/jama.2011.1615

48. Yao Y, Yang Q, Zhong M, He H, Song J. Diagnostic and prognostic value of speckle tracking echocardiography for right ventricular dysfunction in sepsis: a retrospective observational study. BMC Cardiovasc Disord. 2025;25:717. doi:10.1186/s12872-025-05175-9

49. Yu X, Gao J, Zhang C. Sepsis-induced cardiac dysfunction: mitochondria and energy metabolism. Intensive Care Med Exp. 2025;13:20. doi:10.1186/s40635-025-00728-w

50. Zhou X, Pan J, Wang Y, Wang H, Xu Z, Zhuo W. Left ventricular-arterial coupling as a predictor of stroke volume response to norepinephrine in septic shock – a prospective cohort study. BMC Anesthesiol. 2021;21:56. doi:10.1186/s12871-021-01276-y

51. Zhou X, Zhang Y, Pan J, et al. Optimizing left ventricular-arterial coupling during the initial resuscitation in septic shock – a pilot prospective randomized study. BMC Anesthesiol. 2022;22:31. doi:10.1186/s12871-021-01553-w

52. Zhou X, et al. Changes in left ventricular-arterial coupling during resuscitation are associated with mortality in patients with septic shock – a post-hoc analysis of a randomized trial. J Crit Care. 2024;84:154868. doi:10.1016/j.jcrc.2024.154868
